# Single Dose Gonadotropin-Releasing Hormone Agonist Luteal Support in Fresh Embryo Transfer: Variation in Timing, Type, and Dosage

**DOI:** 10.3389/fmed.2022.760430

**Published:** 2022-02-17

**Authors:** Muhammad Azrai Abu, Jojinah Vindah Alexander, Abdul Kadir Abdul Karim, Mohd Faizal Ahmad, Mohd Hashim Omar

**Affiliations:** ^1^Department of Obstetrics and Gynaecology, UKM Medical Centre, Kuala Lumpur, Malaysia; ^2^Department of Obstetrics and Gynaecology, Hospital Wanita Dan Kanak-Kanak Likas, Sabah, Malaysia

**Keywords:** *in vitro* fertilization, luteal phase support, GnRH agonist, single dose administration, fresh embryo transfer (fresh ET)

## Abstract

**Objectives:**

To evaluate the effects of the addition of single-dose GnRH agonist to the routine progestogens use for luteal phase support on IVF outcome as compared to progestogens only.

**Methods:**

This is a retrospective case-control study on selected patients who underwent IVF treatment with fresh embryo transfer (ET) under Medically Assisted Conception Unit, University Kebangsaan Malaysia Medical Center for the period of June 2015–June 2018. A higher dose of 0.2 mg subcutaneous Decapeptyl was administered 2 days before fresh ET concurrent with routine progestogen support. Patients with different luteal phase regimes, frozen embryo transfer and medical records with missing data were excluded. Their medical records were reviewed, and data analyzed. The pregnancy outcomes measured included biochemical pregnancy rates, clinical pregnancy rates, live birth rates and miscarriage rates.

**Results:**

A total of 786 patients were analyzed. Four hundred forty-four patients were given luteal phase support with progestogens and GnRH agonist, whereas 342 patients served as control were given progestogens only. The study group showed higher biochemical pregnancy rate (47.7 vs. 44.4%,), clinical pregnancy rate (25.7 vs. 23.4%) and livebirth rate (24.3 vs. 22.2%), respectively but not statistically significant. The rate of miscarriage among the study group was lower (4.5% vs 9.4%) compared to the progestogen group alone. Nonetheless, the OHSS rate was slightly increased in the study group (4.5 vs. 3.5%) despite using a mild stimulation protocol.

**Conclusions:**

New regime of GnRH agonist luteal support in addition to the standard progestogen support was found to be beneficial in overall IVF outcome.

## Introduction

It is estimated that approximately 15% of the population, or one in six to seven couples in the western world experienced an unwanted delay in conception. Assisted reproduction treatment (ART) enables couples to conceive and ultimately give birth to a healthy baby. The ART includes IUI, IVF and ICSI. The treatment cycle for IVF involves several steps from pituitary down-regulation to ovarian stimulation, ovulation trigger, oocyte collection, luteal phase support, and embryo transfer.

One of the primary concerns was luteal phase deficiency which was described in cycles using pituitary down-regulation with a GnRH agonist, GnRH antagonist and currently with minimal stimulation regime (1, 2). Luteal phase deficiency is characterized by premature regression of the corpus luteum, leading to a shortened luteal phase with evidence of low progesterone levels and delay of the secretory transformation of the endometrium thus leading to poor ART outcome (3). The luteal phase support is a common practice in ART which has been proven to improve ART results by using various routes of progestogens with or without estrogen (4).

Lately, the role of GnRH agonist as luteal phase support has been recommended by various studies though the mechanism is still debatable. It has been postulated that GnRH agonist might support the corpus luteum by stimulating the secretion of luteinizing hormone by pituitary gonadotroph cells, or by acting directly on the endometrium through the local receptor expression (5). However, the optimum dosage and administration time of GnRH agonist with regards to fresh embryo transfer were still debatable.

Therefore, this study was designed to evaluate the effects of a higher dosage of GnRH agonist in luteal phase support 2 days prior to fresh embryo transfer. The biochemical pregnancy rates, clinical pregnancy rates, live birth rates and miscarriage rates between these regimes were compared.

## Methods

### Study Design and Participants

This is a retrospective case-control study on selected patients who underwent IVF treatment under MAC (Medically Assisted Conception) Unit, University Kebangsaan Malaysia Medical Center from June 2015 to June 2018. All patients who gave informed consent allowing the use of their clinical records were included. Their medical records were reviewed, and data were analyzed. They were given progestogens plus GnRH agonist or progestogens only as luteal phase support based on two physician preferences. Patients with different luteal phase support, frozen embryo transfer and medical records with missing data were excluded.

### Study Protocol

All women who underwent control-ovarian stimulation (COH) regime using either gonadotrophin combination with GnRH antagonist protocol or mild stimulation protocol using oral letrozole or clomiphene citrate were included in this study. All women underwent follicle tracking by transvaginal ultrasound until the dominant follicle size was >18 mm. IM Ovidrel® 250 mg (Zuellig Pharma–Merck) was given proceeding with oocyte retrieval (OR) at least 35–36 h later under transvaginal ultrasonography guidance. The 17-gauge single-lumen needles were used for oocyte retrieval under sedation.

ICSI was performed according to local protocol. Zygotes were cultured up to day 5 in G1 medium (Vitrolife). On day 5, embryos were graded according to previously described criteria. One or two embryos were transferred, depending on the morphological score and the developmental stage of the embryo, as well as the age of the patient.

Regardless of the ovarian stimulation protocols, these women were assigned into two groups; progestogens with the addition of GnRH agonist and progestogen-only for luteal phase support. Both groups were given routine progestogen support (Tablet Duphaston 10 mg tds) for 2 weeks duration starting from the day of oocyte retrieval.

In the study group, there was an additional subcutaneous GnRH agonist administrated as a single dose of 0.2 mg Decapeptyl given on day 3 after ICSI. The other group with no GnRH agonist given was the control group.

### Outcome Measures

Pregnancy was diagnosed by measuring serum hCG levels on day 14 after embryo transfer. Positive implantation (biochemical pregnancy) was defined as a serum hCG level >10 mIU/ml. Clinical pregnancy was defined as an ongoing pregnancy with the fetus and a positive heartbeat visualized by ultrasound at 6 weeks of pregnancy. Live birth rate was the birth of a viable fetus beyond 24 weeks of gestation.

### Ethical Consideration

This study was approved by UKM Research Ethics Committee (PPI/111/8/JEP-2018-619).

This study was also registered with ClinicalTrials.gov (NCT04174378).

### Data Analysis and Interpretation

The data analysis was done using SPSS version 23. Descriptive data were expressed as mean+/– standard deviation (SD) or frequencies (no of cases) and percentages when appropriate. The student's *t*-test was used to compare numerical variables between the study groups for an independent sample. Categorical data were analyzed using Chi-square or Fisher's exact test. A value of *P* < 0.05 is considered statistically significant. The data collected were analyzed using an intention to treat basis.

## Results

A total of 786 patients were included. A total of 444 patients were given luteal phase support with progestogens and GnRH agonist, whereas 342 patients served as control were given progestogens only. There was no significant difference between the two groups in terms of demographic characteristics such as age, ethnicity, duration of infertility, type and cause of infertility (Table 1).

**Table 1:** Demographic characteristics of study population (*n* = 786).

**Demographic** **characteristics**	**GnRH agonist (***n*** = 444)**	**Control** **(***n*** = 342)**	***p*** **-Value**
Age (years)	34.8 ± 4.4	35.3 ± 4.6	0.206
Duration of infertility (years)	5.39 ± 3.03	5.35 ±3.22	0.834
Type of infertility			0.056
Primary (%)	304(68.5%)	256 (74.9%)	
Secondary (%)	140 (31.5%)	86 (25.1%)	
Cause of infertility			0.099
Male factor (%)	128 (28.8%)	128 (37.4%)	
Endometriosis (%)	82 (18.5%)	74 (21.6%)	
PCOS (%)	56(12.6%)	18 (5.3%)	
Unexplained (%)	70 (15.8%)	42 (12.3%)	
Tubal factor (%)	72 (16.2%)	50 (14.6%)	
Anovulation (%)	36 (8.1%)	30 (8.8%)	
Ethnicity			0.511
Malay (%)	330 (74.3%)	274 (80.1%)	
Chinese (%)	78 (17.6%)	42 (12.3%)	
Indian (%)	26 (5.9%)	20 (5.8%)	
Others (%)	10 (2.3%)	6 (1.8%)	

*Continuous data are expressed in mean ± standard deviation. Categorical data are expressed in number and percentage in parenthesis*.

Most patients in the GnRH agonist group used a mild stimulation regime compared to the control group (60.8 vs. 21.1%). Meanwhile, the duration of ovarian stimulation was similar between both groups (11.6 vs. 11.9) days. The number of retrieved oocytes and mature oocytes were lower in the study group (10.2 ± 6.5 vs. 11.2 ± 6.8) and (8.0 ± 5.5 vs. 8.9 ± 5.5), respectively. Whereas, the number of embryo transfers (1.8 ± 0.7 vs. 1.7 ± 0.6) and endometrial thickness (11.0 ± 1.5 vs. 11.1 ± 1.7) did not differ between the groups (Table 2).

**Table 2:** Sub-analysis ovarian stimulation protocol.

**Outcome**	**GnRH agonist ***n*** (%)**	**Control** ***n*** **(%)**	***p*** **-Value**
Duration of stimulation (*n* = days)	11.6 ± 1.4	11.9 ± 1.2	0.11
COH regime:		
Gonadotrophin protocol combination with GnRH antagonist	174 (39.2%)	270 (78.9%)	<0.001*
Mild stimulation regime	270 (60.8%)	72 (21.1%)	
No oocytes retrieved	10.2 ± 6.5	11.2 ± 6.8	0.034
No mature oocytes	8.0 ± 5.5	8.9 ± 5.5	0.024
No embryo transferred	1.8 ± 0.7	1.7 ± 0.6	0.06
ET thickness during embryo transfer	11.0 ± 1.5	11.1 ± 1.7	0.112

*Continuous data are expressed in mean ± standard deviation. Categorical data are expressed in number and percentage in parenthesis*.

* *Statistically significant difference between groups, with p < 0.05*.

The GnRH agonist group showed higher biochemical pregnancy rates (47.7 vs. 44.4%,), clinical pregnancy rates (25.7 vs. 23.4%) and live birth rates (24.3 vs. 22.2%) however it was not statistically significant. Furthermore, the rate of miscarriage among the GnRH agonist group was lower (4.5 vs. 9.4%) compared to control. With regards to the complications, the OHSS rate was comparable between GnRH group vs. control (4.5 vs. 3.5%) and the difference was not statistically significant (Table 3).

**Table 3:** Comparison of pregnancy outcomes between GnRH agonist and control group.

**Outcome**	**GnRH agonist ***n*** (%)**	**Control** ***n*** **(%)**	**OR (95% CI) ***p***-value**
Biochemical pregnancy (*n* = 364)	212 (47.7%)	152 (44.4%)	1.14 (0.86–1.51) 0.38
Clinical pregnancy (*n* = 194)	114 (25.7%)	80 (23.4%)	1.13 (0.81–1.57) 0.50
Live birth (*n* = 184)	108 (24.3%)	76 (22.2%)	1.12 (0.80–1.57) 0.498
Miscarriage (*n* = 52)	20 (4.5%)	32 (9.4%)	0.45 (0.25–0.81) 0.009*
OHSS rate	20 (4.5%)	12 (3.5%)	1.29 (0.625–2.69) 0.58

*Data presented as n (%), analyzed using fisher exact test, difference between two groups expressed as odds ratio (OR) and 95% confidence interval (CI)*.

* *Statistically significant difference between groups, with p < 0.05*.

Subsequently, pregnancy outcomes were further categorized based on age factor whereby it was divided into age below and above 35 years old. Those with age more than 35 years old and given GnRH agonist in the luteal phase, had higher biochemical pregnancy, clinical pregnancy and live birth rates compared to the control group. However, it is not statistically significant. The miscarriage rate was higher in the control group (Table 4).

**Table 4:** Pregnancy outcomes between GnRH agonist and control group following women age.

	**Women age <35 years old**			**Women age >35 years old**		
**Outcome**	**GnRH agonist (***n*** = 216)**	**Control** **(***n*** = 150)**	**OR (95% CI) ***p***-value**	**GnRH agonist** **(***n*** = 228)**	**Control (***n*** = 192)**	**OR (95% CI) ***p***-value**
Biochemical pregnancy *n* (%)	106 (49.1%)	68 (45.3%)	1.16 (0.76–1.76) 0.52	106 (46.5%)	84 (43.8%)	1.11(0.75–1.64) 0.62
Clinical pregnancy *n* (%)	62 (28.7%)	44 (29.3%)	0.97 (0.61–1.53) 0.90	52 (22.8%)	36 (18.8%)	1.28 (0.79–2.06) 0.33
Live birth *n* (%)	60 (27.8%)	44 (29.3%)	0.92 (0.58–1.46) 0.81	48 (21.1%)	32 (16.7%)	1.33 (0.81–2.18) 0.26
Miscarriage *n* (%)	12 (5.6%)	16 (10.7%)	0.49 (0.22–1.07) 0.07	8 (3.5%)	16 (8.3%)	0.40 (0.16–0.95) 0.03*

*Data presented as n (%), analyzed using fisher exact test, difference between two groups expressed as odds ratio (OR) and 95% confidence interval (CI)*.

* *Statistically significant difference between groups, with p < 0.05*.

Different ovarian stimulation protocols have a different impact on pregnancy outcomes. Those who underwent a mild stimulation regime have a significantly lower biochemical pregnancy rate in the GnRH agonist-luteal phase compared to control. However, the clinical pregnancy rate and live birth rate were higher than non-GnRH agonist group. The miscarriage rate was significantly lower in the study group as well. In patients who underwent gonadotrophin combination with GnRH antagonist, the biochemical pregnancy rate was higher in the study group and it was statistically significant. The clinical pregnancy and live birth rates were higher with a lower miscarriage rate compared to the control group but not statistically significant (Table 5).

**Table 5:** Different ovarian stimulation protocols and pregnancy outcome.

	**Gonadotrophin combination with GnRH antagonist stimulation protocol (*****n*** **= 444)**			**Mild stimulation regime (*****n*** **= 342)**		
**Outcome**	**GnRH agonist (***n*** = 174)**	**Control** **(***n*** = 270)**	**OR (95% CI) ***p***-value**	**GnRH agonist** **(***n*** = 270)**	**Control (***n*** = 72)**	**OR (95% CI)** ***p*****-value**
Biochemical pregnancy *n* (%)	92 (52.9%)	102 (37.8%)	1.85 (1.26–2.72) 0.002*	120 (44.4%)	50 (69.4%)	0.35 (0.20–0.61) <0.001*
Clinical pregnancy *n* (%)	52 (29.9%)	68 (25.2%)	1.27 (0.82–1.93) 0.28	62 (23%)	12 (16.7%)	1.49 (0.75–2.95) 0.33
Live birth *n* (%)	50 (28.7%)	64 (23.7%)	1.29 (0.84–1.99) 0.27	58 (21.5%)	12 (16.7%)	1.37 (0.69–2.71) 0.415
Miscarriage *n* (%)	10 (5.7%)	22 (8.1%)	0.69 (0.31–1.49) 0.45	10 (3.7%)	10 (13.9%)	0.23 (0.10–0.60) 0.003*

*Data presented as n (%), analyzed using fisher exact test, difference between two groups expressed as odds ratio (OR) and 95% confidence interval (CI)*.

* *Statistically significant difference between groups, with p < 0.05*.

## Discussion

Several previous studies have shown the positive effect of GnRH agonist administration during the luteal phase in the IVF cycle (6–11). There was no specific mechanism on its action but has potential in maintaining corpus luteum activities with the expression of the endometrial receptor for implantation. The landmark paper by Tesarik et al. utilizing GnRH agonist as luteal phase support 6 days after ICSI in fresh transferred cycle revealed a significant improvement in the implantation and live birth rate (12). Therefore, the modification in the dosage, type and time of administration of the GnRH agonist in this study needs to be evaluated on its IVF outcome compared to the routine progestogens only use for luteal phase support.

The majority of previous studies used 0.1 mg of GnRH agonist for luteal phase support and have shown significant benefit in the overall IVF outcome. Davar et al. (13) reported a significant increase in the clinical pregnancy rate (26 vs. 21%) and Zafardoust et al. (14) also managed to achieve a biochemical pregnancy rate up to 32.6% with a similar dosage of GnRH agonist. A doubling in the GnRH agonist dosage up to 0.2 mg in our study population showed a higher biochemical pregnancy rate (47.7 vs. 44.4%,), clinical pregnancy rate (25.7 vs. 23.4%) and live birth rate (24.3 vs. 22.2%) but there was no significant doubling in the overall outcome. With regards to the time of GnRH agonist administration, there was a variety of luteal phase protocols available. Benmachiche et al. (15) and Razieh et al. (8) administered GnRH agonist 6 days after oocyte retrieval resulting in a higher clinical pregnancy rate (38 vs. 31%) and (25.5 vs. 10.0%), respectively but not significant statistically. Besides that, Qublan et al. (7) achieved similar pregnancy outcomes with different GnRh agonist regimes given at embryo transfer and 3 days later. Taking into consideration the early luteal defect following oocyte retrieval, our study population received GnRH agonist as early as 3 days following ICSI. We achieved a higher clinical pregnancy rate although it was not statistically significant which is consistent with previous studies (7, 15).

Several previous studies used different types of GnRH agonist and demonstrated similar findings to our study. Pirard et al. conducted a randomized controlled trial using intranasal GnRH agonist and found a similar higher biochemical and clinical pregnancy rate (16, 17). Subsequently, randomized control trial by Fujii et al. using daily intranasal GnRH agonist up to 14 days following embryo transfer revealed a higher clinical pregnancy rate (44.5 vs. 34.3%) and live birth rate (23.6 vs. 15.7%) (18). On the other hand, Isik et al. demonstrated that with the addition of single-dose GnRH agonist (SC Leuprolide acetate 0.5 mg) on day 6 after ICSI, there were significantly higher clinical pregnancy rates (40.5 vs. 20%) and live birth rates (35.1 vs. 16.3%) (19).

The majority of patients in our study group used a mild stimulation regime as compared to previous studies which have shown benefits with GnRH antagonist protocol (17, 20) or long GnRH agonist protocol (15, 21). These findings are consistent with the conclusion of Tesarik et al. (5) that regardless of the type of ovarian stimulation protocol used, luteal-phase GnRH agonist group showed significant increase in implantation rate, clinical pregnancy rates and live birth rates. Previous studies also showed a preference in using GnRH agonist as luteal phase support in GnRH agonist triggered IVF cycles (15) due to its detrimental effect on corpus luteum thus affecting the overall pregnancy outcome. However, the use of HCG as a triggering agent in our study does show benefit in the clinical pregnancy outcome parallel with the study by Tesarik et al. (5).

In our study, the rate of miscarriage in GnRH agonist group was low compared to the control (4.5 vs. 9.4%) and statistically significant. These findings were supported by Qublan et al. who also reported a low miscarriage rate (5%) in GnRH agonist group and 8.3% in the control group (7). This prospective study, however, focused on women with endometrial thickness <7 mm which differs from our study population's mean endometrial thickness of 11.5 mm. The higher rate of miscarriage in the IVF cycle is therefore independently related to endometrial thickness as suggested by Chen et al. (22) concerning more toward endometrial pattern or maturity.

A prospective study by Ye et al. has highlighted the importance of age as a significant determining factor influencing the overall pregnancy outcome in all women who underwent frozen embryo transfer (23). Patients who were above 35 years old has significantly higher implantation rates (45.3 vs. 27.8%, *p* < 0.05) following GnRH support. On the contrary, our study showed no significant difference in the total outcome for women age below and above 35 years old following IVF. However, additional GnRH agonist in older women could improve embryonic development and endometrial receptivity, a higher clinical pregnancy rate (22.8 vs. 18.8%) was achieved despite reversal outcome in younger women (28.7 vs. 29.3%).

With regards to complications, the OHSS rate was comparable between the GnRH group vs. control (4.5 vs. 3.5%) and the difference was not statistically significant. This finding was supported in another study by Yildiz et al. with a reported rate of OHSS 5 vs. 5.3% (6). The study by Benmachiche et al. also supports our findings in which there was no difference in OHSS rate (0.6% in both groups) (15).

The study design has its limitation being a retrospective study hence there is a gap in the number of the participants in the treated group and control group. The main confounding factor in our study is the routine progestogen used as luteal phase support and different types of control ovarian stimulation regimes. As this study involves various ethnicity, this data can be applied to the general population in Malaysia.

In conclusion, GnRH agonist is beneficial to be used in addition for luteal phase support as it showed higher biochemical pregnancy, clinical pregnancy and live birth rate and reduced miscarriage rate. Hence, future randomized control trials with a larger sample size should be conducted in our population to find consensus on the best luteal phase support regime.

## Data Availability Statement

The raw data supporting the conclusions of this article will be made available by the authors, without undue reservation.

## Ethics Statement

The studies involving human participants were reviewed and approved by Research and Ethical Committee, Faculty of Medicine, University Kebangsaan Malaysia. The patients/participants provided their written informed consent to participate in this study.

## Author Contributions

MAb and JA: data curation and analysis, writing–drafting, review, and editing. AA: conceptualization and methodology. MAh: project administration. MO: supervision. All authors contributed to the article and approved the submitted version.

## Conflict of Interest

The authors declare that the research was conducted in the absence of any commercial or financial relationships that could be construed as a potential conflict of interest.

## Publisher's Note

All claims expressed in this article are solely those of the authors and do not necessarily represent those of their affiliated organizations, or those of the publisher, the editors and the reviewers. Any product that may be evaluated in this article, or claim that may be made by its manufacturer, is not guaranteed or endorsed by the publisher.
